# Lifestyle Interventions to Tackle Cardiovascular Risk in Thyroid Hormone Signaling Disorders

**DOI:** 10.3390/nu17132053

**Published:** 2025-06-20

**Authors:** Simone Rodolfi, Giuditta Rurale, Federica Marelli, Luca Persani, Irene Campi

**Affiliations:** 1 Department of Medical Biotechnology and Translational Medicine, University of Milan, 20100 Milan, Italy; simone.rodolfi@unimi.it; 2 IRCCS Istituto Auxologico Italiano, Department of Endocrine and Metabolic Diseases, 20100 Milan, Italyfederica.marelli.fm@gmail.com (F.M.)

**Keywords:** resistance to thyroid hormone β (RTHβ) and α (RTHα), monocarboxylate transporter 8 (MCT8) defects, selenoprotein deficiency, cardiovascular risk, lifestyle, physical exercise, nutrition, diet

## Abstract

Thyroid hormones (THs) play a central role in cardiovascular and metabolic regulation, influencing lipid metabolism, insulin sensitivity and resting energy expenditure. Inherited disorders of impaired sensitivity to THs—including resistance to thyroid hormone alpha (RTHα) and beta (RTHβ), monocarboxylate transporter 8 (MCT8) deficiency and selenoprotein deficiency—lead to complex, multisystemic clinical features. Although these conditions are rare, with RTHβ being the most common and affecting about 1 in 20,000 newborns, they share clinical features with more prevalent thyroid disorders, such as hypothyroidism and hyperthyroidism, as well as neurological manifestations including muscle wasting and spasticity. These conditions present abnormal patterns of thyroid function and are associated with tissue-specific comorbidities such as arrhythmias, heart failure, dyslipidemia, hepatic steatosis, insulin resistance, and metabolic syndrome. To date, no targeted or controlled studies have evaluated the impact of lifestyle modifications in these patient populations. Therefore, this narrative review proposes plausible management strategies based on pathophysiological insights into the effects of thyroid hormones on target organs, combined with clinical reasoning and evidence extrapolated from related disorders. Physical exercise and diet may complement pharmacological treatments (e.g., levothyroxine or TRIAC) to improve cardiovascular and metabolic outcomes. In RTHβ, aerobic exercise enhances cardiovascular health, while a Mediterranean diet supports lipid control and glycemic parameters. In RTHα, physical exercise may aid neuromotor development, and a fluid-rich, fiber-moderated diet can alleviate constipation. In MCT8 deficiency, physiotherapy may improve mobility and relieve contractures, while nutritional support (e.g., feeding tube, gastrostomy) can be necessary to tackle feeding difficulties and reduce pulmonary complications. In selenoprotein deficiency, low-to-moderate physical exercise and an antioxidant-rich diet may protect against oxidative stress at several tissue levels. Although quantitative evidence is limited, this narrative review synthesizes current insights, providing a meaningful basis for future validation and research.

## 1. Introduction

The aim of this review is to describe the impact on the cardiovascular (CV) and metabolic system of rare disorders of impaired sensitivity to thyroid hormones (THs). These monogenic conditions can affect the transport, metabolism and action of THs in cells, and are characterized by discordant thyroid function patterns associated with multisystemic effects (Table 1), reflecting the pleiotropic involvement of THs in physiological regulation [1]. We will start this discussion with a comprehensive description of the factors involved in the regulation of the hypothalamus–pituitary–thyroid axis and the impact of conventional primary thyroid dysfunction on target organs. This provides a necessary framework to better understand the complex pathophysiology and shared mechanisms underlying these rare multisystem disorders, in which signs of both hypo- and hyperthyroidism may coexist. Then, we will examine the existing knowledge regarding cardiometabolic risk in patients with inherited disorders of thyroid signaling, and we will highlight or propose the potential role of physical exercise and dietary interventions in primary prevention, as these aspects remain largely underexplored.

## 2. Materials and Methods

We performed a narrative literature review using PubMed and Google Scholar, including articles published in English up until April 2025. We prioritized high-relevance and high-quality articles, particularly those authored by expert groups in the field of rare thyroid hormone signaling disorders. Three authors (SR, IC and GR) independently conducted the literature search and screened titles and abstracts for relevance. Any disagreements regarding study inclusion were resolved through discussion and consensus. The search strategy combined keywords and MeSH terms using Boolean operators (AND, OR), and was structured to reflect the main sections of the review. To explore the physiological and pathological effects of thyroid hormones on peripheral tissues, we combined terms such as “hypothyroidism”, “hyperthyroidism”, “thyroid dysfunction” and “thyroid hormones” with “heart”, “cardiovascular system”, “CV system”, “skeletal muscle,” “liver,” “adipose tissue”, “WAT”, “BAT”, “browning” and “REE”. We also investigated the relationship between thyroid dysfunction and lifestyle factors using combinations such as “thyroid dysfunction” AND “exercise” or “diet.” In the second part of the review, we focused on rare disorders of thyroid hormone signaling. For this, we combined disease-specific terms with outcomes related to cardiovascular risk and metabolism and preventive interventions. Reference lists of selected articles were also manually screened to ensure comprehensive coverage of the topic. The following filters were applied: publication date between 1995 and 2025, classical article, clinical study, clinical trial (phase I, II, III, IV), controlled clinical trial, guideline, meta-analysis, multicenter study, observational study, randomized controlled trial, review, systematic review, English, humans, and exclusion of preprints. Additionally, it should be noted that for the description of the syndromes, the temporal filter was removed, and therefore, articles from 1975 onward were included.

## 3. Hypothalamus–Pituitary–Thyroid Axis

The hypothalamus-pituitary-thyroid axis (HPTA) is a regulatory system involving negative feedback loops from the hypothalamus to the pituitary and to the peripheral target glands [2]. Mediators of this complex neuroendocrine system include thyrotropin-releasing hormone (TRH), thyrotropin (TSH), THs and their specific receptors (TRHR, TSHR and thyroid hormone receptors). TRH, secreted by the paraventricular nucleus (PVN) of the hypothalamus, stimulates the pituitary gland to release TSH. Upon binding to its receptor (TSH-R), TSH stimulates the synthesis and release of THs and supports the growth of the thyroid gland.

**Figure 1:**
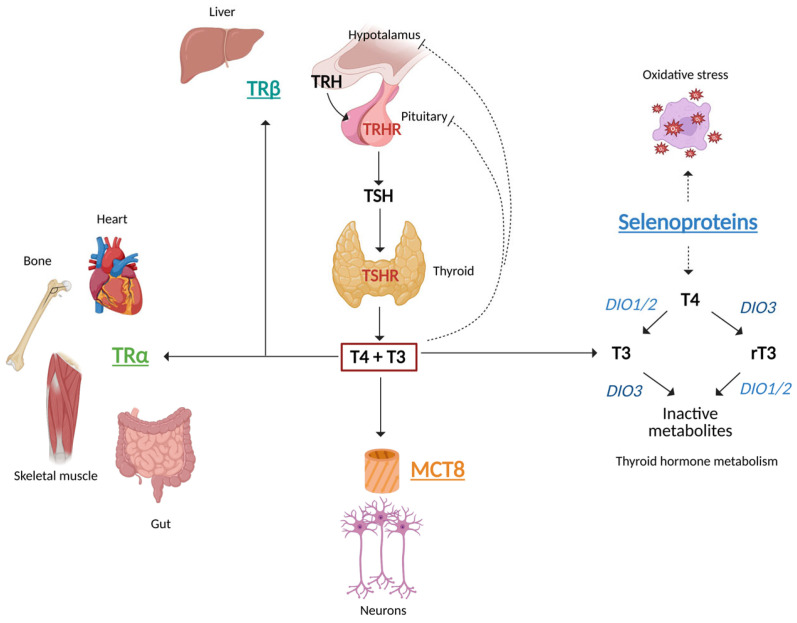
The hypothalamus–pituitary–thyroid axis (HPTA) and mechanism involved in disorders of impaired sensitivity to thyroid hormones (THs). THs, thyroxine, T4, and triiodothyronine, T3, bind to nuclear TH receptors (TRs), which are present in two isoforms, TRα and TRβ, encoded by two different genes, *THRA* and *THRB*, respectively. Genetic mutations in *THRA* and *THRB* cause two different syndromes, called resistance to thyroid hormone alpha (RTHα) and beta (RTHβ). MCT8, encoded by the *SLC16A2* gene, is essential for the uptake of T3 into neurons. Loss-of-function mutations in *SLC16A2* result in Allan–Herndon–Dudley syndrome. Selenoproteins reduce antioxidative stress and regulate TH metabolism, assisting the activity of deiodinases. Mutations in genes involved in selenoprotein synthesis cause selenoprotein deficiency. Legend: TRH, thyrotropin-releasing hormone; TRHR, thyrotropin-releasing hormone receptor; TSH, thyroid stimulating hormone; TSHR, thyroid stimulating hormone receptor; T4, thyroxine; T3, triiodothyronine; MCT8, Monocarboxylate Transporter 8; TRα, thyroid hormone receptor α; TRβ, thyroid hormone receptor β; DIO, iodothyronine deiodinase.

THs regulate several physiological processes, including neurodevelopment, fertility, metabolism, thermogenesis, heart rate, gut motility, growth and skeletal development [3]. To exert their actions, THs bind to nuclear TH receptors (TRs), which exist in two isoforms, TRα and TRβ, encoded by two different genes, *THRA* and *THRB*, respectively (Figure 1). TRα is predominantly expressed in the heart, skeletal muscles and brain, while TRβ is primarily found in the liver, kidney, sensorineural tissues, hypothalamus and pituitary. Functional TRs are composed of heterodimers with the retinoid X receptor (*RXR*) or, less frequently, exist as homodimers. After triiodothyronine (T3) binding, TRs translocate into the nucleus and act as activators or repressors of gene transcription, upon binding to specific thyroid-responsive elements (TREs) located in the promoters of responsive genes. TRβ, expressed in the pituitary, is the main regulator of the negative feedback, as *TSHB*, which encodes the beta subunit of TSH, is downregulated by THs (Figure 1). Genetic mutations in *THRA* and *THRB* cause two different syndromes, called resistance to thyroid hormone alpha (RTHα) and beta (RTHβ), also known as Refetoff Syndrome [4] (Figure 1). The different biochemical patterns of these syndromes (Table 1) are the direct consequence of the tissue distribution of TRs, as described above.

## 4. Thyroid Hormone Synthesis, Metabolism and Action

The thyroid follicles are the functional units of the thyroid gland. These spherical structures are composed of a wall of follicular cells (thyrocytes), delimiting a central lumen filled with colloid. Each thyrocyte has two poles: a basolateral membrane that faces the interfollicular connective tissue and an apical membrane that contacts the colloid. The main component of the colloidal fluid is the dimeric glycoprotein thyroglobulin (Tg) produced in the endoplasmic reticulum (ER) of the thyrocytes [5].

Upon TSH stimulation, the sodium/iodide symporter (NIS) located in the basolateral membrane of the thyrocyte actively transports iodide from the bloodstream into the cytoplasm. Iodide is then oxidized by thyroid peroxidase (TPO) located in the apical pole and incorporated into the aromatic rings of tyrosine residues of Tg, leading to the synthesis of monoiodotyrosine (3′-MIT) or diiodotyrosine (3, 5-DIT). TPO also catalyzes the coupling of these iodotyrosines, which are linked by an ether bond. This results in the synthesis of two THs: 3,5,3′,5′ thyroxine (T4) (DIT + DIT) and 3,3′,5 triiodothyronine (T3) (MIT + DIT), which are then released into the circulation by endocytosis and subsequent proteolytic cleavage from Tg [3].

Once secreted, THs are transported from the thyroid to target tissues by albumin, thyroxine-binding globulin and transthyretin. TH transport across the cellular membranes is mediated by specific transporters, including the monocarboxylate transporter 8 (MCT8) encoded by the *SLC16A2* gene. MCT8 is essential for the uptake of T3 into neurons. Therefore, loss-of-function mutations in the *SLC16A2* gene result in the severe neurological phenotype of Allan–Herndon–Dudley syndrome [6]. Once inside target cells, T3 binds to nuclear TRs, modulating gene expression (Figure 1). The intracellular actions of THs are finely regulated by activating and inactivating enzymes called deiodinases, which remove iodine atoms from TH (deiodination). There are three deiodinases: type 1 (D1, encoded by the *DIO1* gene), type 2 (D2, encoded by the *DIO2* gene) and type 3 (D3, encoded by the *DIO3* gene). D1 catalyzes both outer-ring deiodination (ORD) and inner-ring deiodination (IRD), whereas D2 and D3 catalyze only ORD and IRD, respectively [7].

T4, which is the predominant circulating TH, is a prohormone, while the biologically active hormone is T3. Only 20% of circulating T3 is directly secreted from the thyroid, whereas 80% is produced in peripheral tissues as a result of ORD of T4. Conversely, IRD generates the inactive form reverse-T3 (rT3). T3 is subsequently inactivated by IRD and rT3 by ORD, with T2 produced in both cases. Further degradation of THs occurs via glucuronidation, deamination and sulfation [7]. The availability of THs regulates the expression of deiodinases, with D1 and D3 being upregulated and D2 downregulated by T3. Selenium and iodine are two critical trace elements involved in TH metabolism. Selenium is the cofactor of deiodinases, while iodine is essential for TH synthesis. In addition, iodine availability modulates DIO activity itself, since iodine deficiency downregulates D1 and D3 and upregulates D2. Mutations in genes involved in selenoprotein synthesis cause selenoprotein deficiency [8,9], leading to alterations in thyroid function and increased susceptibility to oxidative stress (Figure 1).

## 5. Primary Thyroid Dysfunction and the CV System

THs modulate heart function through both genomic and nongenomic mechanisms [10]. THs modulate the expression of genes encoding ion channels, structural proteins, ATPases, amino acids and glucose transporters, as well as adrenergic receptors, therefore modulating heart rate (HR), cardiac output, and blood pressure [5]. In addition, THs reduce peripheral vascular resistance and decrease kidney perfusion with upregulation of the renin–angiotensin–aldosterone system (RAAS) [11].

Overt hyperthyroidism is associated with several time-dependent CV alterations. In the earlier stages, hyperthyroidism improves left ventricular mechanical efficiency, due to the increased synthesis of heavy myosin chain proteins, hyperdynamic circulation, increased cardiac preload and improved energy metabolism [12]. Conversely, long-term untreated hyperthyroidism causes high-output heart failure (HF), characterized by increased left ventricular mass, increased left atrial size, diastolic dysfunction pulmonary hypertension and tachycardia [12,13,14]. In addition, upregulation of the RAAS causes fluid retention, leading to liver congestion and peripheral and lung edema [11]. In a minority of patients, end-stage dilated cardiomyopathy may occur, which is a tachycardia-induced cardiomyopathy [12].

Increased left atrial pressure, tachycardia-induced ischemia and enhanced atrial ectopic activity contribute to the occurrence of supraventricular arrhythmias (atrial fibrillation—AF—and atrial flutter) in hyperthyroid patients [15,16]. The loss of sinus rhythm is an important contributor to HF in hyperthyroidism.

For these reasons, overt primary hyperthyroidism is associated with an increased risk of CV diseases (CVDs) (AF, major CV events—MACEs, and HF) and all-cause mortality [17,18,19,20]. The impact of subclinical hyperthyroidism is less clear [21,22]. Nevertheless, in euthyroid individuals, fT4 levels in the upper quartile of the normal range are related to all-cause mortality, decreased life expectancy, incident CVDs, sudden cardiac death, and AF [23,24].

Overt primary hypothyroidism induces opposite changes in CV function. In the early stages, hypothyroidism causes left ventricular diastolic dysfunction with preserved systolic function. Long-term overt hypothyroidism also impairs left ventricular function, leading to low cardiac output [25]. However, HF usually occurs only in patients with underlying CV diseases [26]. Rarely, severe hypothyroidism may result in cardiac tamponade due to protein-rich pericardial effusion.

Furthermore, overt primary hypothyroidism is associated with impaired endothelial function [27,28], dyslipidemia, increased insulin resistance and hypertension [29]. Magnetic resonance studies [30,31] have highlighted that hypothyroid patients have increased left ventricular mass with significant myocardial stiffness. Regarding the risk of arrythmias, hypothyroidism causes sinus bradycardia, low voltages and a prolonged QT interval, which may increase the risk of atrioventricular blocks or ventricular arrhythmias, such as torsades de pointes [13]. Despite an increased prevalence of risk factors for arrhythmias such as atherosclerosis, hypertension and myocardial stiffness, epidemiological studies fail to demonstrate an increased prevalence of AF in hypothyroid patients [32,33]. Although, an epidemiological association between subclinical hypothyroidism, impaired endothelial function [27,28], dyslipidemia, increased insulin resistance, hypertension and left ventricular dysfunction have been reported [34,35], the significance of the impact of hypothyroidism on CVDs and CV mortality remains undefined [36].

## 6. Thyroid Dysfunction and Muscles

Several functions of the skeletal muscles are modulated by THs, including metabolic rate and resting oxygen consumption [37]. The intracellular availability of T3 is regulated in skeletal muscles by TH transporters (MCT8 and MCT10) and deiodinases (D2 and D3) [37,38,39]. T3 favors the shift from slow-twitch skeletal fibers (type 1) to fast-twitch fibers (type 2), with faster contractile function and better glycolytic and oxidative capacities. This results in increased heat dissipation and energy expenditure [40]. This phenotypic change in the muscle fibers relies on the increased expression of genes regulating myosin chain synthesis, such as myogenic regulatory factors (MRFs), myogenin and myoblast determination protein 1 (MYOD1), and muscle metabolism, such as fast-twitch isoforms of sarcoplasmic reticulum Ca-ATPase (SERCA1a), uncoupling protein 3 (UCP3), glucose transporter GLUT-4 (SLC2A4), NADP-dependent malic enzyme (ME1) and the mitochondrial protein muscle glycerol-3-phosphate dehydrogenase (mGPDH). On the contrary, myosin-7 (MYH7), calcineurin and the slow-twitch isoforms of sarcoplasmic reticulum Ca-ATPase (SERCA2a) are downregulated by T3 [37].

Therefore, overt hyperthyroidism increases the basal metabolic rate and enhances protein turnover, leading to muscle wasting and impaired functional exercise capacity compared to controls [41]. Muscle damage is secondary to elevated mitochondrial metabolism and decreased glutathione peroxidase activity [42], with functional impairment occurring not only in proximal and distal skeletal muscles [43], but also in the diaphragmatic/intercostal muscles [44], contributing to exertional dyspnea. These anomalies are reversible with the restoration of euthyroidism [45], but a longer disease duration and lower BMI are associated with a worse muscle phenotype [46] and a longer recovery.

Conversely, overt hypothyroidism causes a different myopathy, characterized by slower contraction and relaxation with increased muscle stiffness. The key feature of hypothyroid myopathy is oxidative damage, due to aberrant glycogen metabolism and altered oxidative metabolism within the actin–myosin units. Its manifestations are highly variable, ranging from asymptomatic mild-to-moderate CK elevation to rhabdomyolysis [47]. The most frequent complaints reported by hypothyroid patients are muscle pain, weakness and cramps [48,49].

## 7. Thyroid Dysfunction and the Liver

The action of THs in the liver explains many of their metabolic effects. In addition, the liver also regulates circulating TH levels, being involved in T3 synthesis and rT3 clearance by D1 activity, as well as in T3 inactivation via D3. Moreover, the liver synthesizes TH transport proteins, including albumin, transthyretin and TBG.

THs influence lipid mobilization and degradation via hormone-sensitive lipases, as well as fatty acid β-oxidation, cholesterol synthesis, bile acid synthesis and lipoprotein homeostasis [50,51]. T3 reduces serum apolipoprotein B100 and increases the activity of cholesteryl ester transferase (CEPT) and hepatic lipase. Furthermore, T3 promotes the hepatic reuptake of LDL, the synthesis and release of free fatty acids (FFAs) in peripheral tissues and their hepatic uptake. THs also regulate carbohydrate metabolism, enhancing intestinal glucose absorption, glycogenolysis and gluconeogenesis from lactate, aminoacids and glycerol [52]. Finally, THs are involved in the organization of hepatic microtubules through upregulation of β-tubulin via TRβ [53].

Hypothyroid patients often exhibit dyslipidemia, with increased serum levels of total and LDL cholesterol and triglycerides [54,55], while opposite changes are observed in hyperthyroid individuals. Liver steatosis is another metabolic consequence of hypothyroidism, since reduced lipolysis and cholesterol clearance result in the accumulation of LDL and triglycerides in the liver [56]. Furthermore, hypothyroidism is associated with insulin resistance, contributing to excessive triglyceride accumulation in hepatocytes [55]. Finally, impaired hepatic autophagy is an additional potential mechanism that explains liver steatosis associated with hypothyroidism [56]. Interestingly, in a retrospective series of 103 patients with metabolic dysfunction-associated fatty liver disease (MASLD), hypothyroidism was highly prevalent (15.5%). However, hypothyroidism did not correlate with the severity of steatosis, but with an increased HOMA index suggestive of insulin resistance and triglyceride levels >150 mg/dL [57].

Conversely, hyperthyroidism is associated with increased lipolysis and enhanced expression of glucose-6-phosphatase, malic enzyme and squalene monooxygenase [50]. Moreover, T3 administration to patients with MASLD significantly reduced triglyceride accumulation in the liver [58]. Such therapy is not justified due to the severe adverse effects of iatrogenic hyperthyroidism. For this reason, selective TR-β agonists have been developed. While some agonists have been discontinued due to severe side effects, in 2024, Resmetirom, an oral TR-β agonist, was finally approved by the FDA for the treatment of adults with MASLD associated with liver fibrosis [59].

## 8. Thyroid Dysfunction and Adipose Tissue

TH significantly influences the functions of adipose tissue and its histological phenotype. T3 enhances adenosine triphosphate (ATP) utilization, promotes mitochondrial biogenesis and stimulates thermogenic pathways, especially in brown adipose tissue (BAT) [60,61]. This thermogenic effect of T3 depends on the upregulation of uncoupling protein 1 (UCP1), which uncouples oxidative phosphorylation from ATP production. THs also promote the “browning” of white adipose tissue (WAT), whereby WAT acquires BAT-like thermogenic features, although the clinical significance of this in humans remains unclear [62,63]. In addition, T3 increases the core body temperature and stimulates appetite and the activity of the sympathetic central system by acting directly on the ventromedial nucleus of the hypothalamus (VMH), which, in turn, activates BAT thermogenesis [64]. THs also modulate circulating levels of adipokines and myokines, such as irisin, FGF21, fetuin A, and neuregulin 4 (NgL-4), that are involved in the peripheral regulation of resting energy expenditure (REE) and lipid metabolism [65,66,67,68]. Interestingly, preadipocytes and adipocytes also express TSHR, and in vitro findings suggest that TSHR activation enhances adipogenesis and favors BAT formation [69].

## 9. Impact of Physical Exercise on CV Risk and Thyroid Function

The WHO defines physical activity (PA) as any bodily movement produced by skeletal muscles that requires energy expenditure and promotes actions to address physical inactivity [70]. There are two types of PA: aerobic (dynamic activities resulting in substantial increases in heart rate and energy expenditure) and anaerobic training (activities aimed at increasing muscular strength and power).

Although the benefits of PA are variable depending on ethnicity, age, genetic factors, food intake and concomitant chronic diseases [71], it is unquestionable that regular PA may prevent several noncommunicable diseases, such as heart disease, hypertension, stroke, diabetes and cancers. PA improves body composition [72,73], maintains/increases muscle mass, reduces the risk of age-related sarcopenia [74], enhances oxygen consumption and cardiorespiratory capacity and improves insulin sensitivity [75,76,77], independently of weight loss [78]. Moreover, PA promotes WAT browning by increasing FGF21 and irisin secretion from skeletal muscles [79] and stimulating additional metabolic pathways mediated by several intermediate metabolites (e.g., lactate, ketone bodies, succinate and kynurenic acid) [80]. The modulation of autonomic tone with enhanced vagal tone and reduced circulating catecholamines explains the reduction in resting HR and the faster HR recovery induced by PA [81]. This is associated with improved endothelial function, reduced angiotensin II levels and increased bioavailability of nitric oxide (NO). Finally, PA modulates the immune system, downregulating pro-inflammatory cytokines (e.g., TNF-α and IL-6) and decreasing serum inflammatory markers (e.g., reactive C protein and homocysteine) [82].

Given these pleiotropic actions of exercise, an influence on thyroid function could reasonably be expected [83]. However, experimental findings in healthy controls are inconsistent, with some reports demonstrating no significant changes and others showing an increase or decrease in THs (mainly T3 and rT3) following PA [84,85,86,87,88,89]. This variability likely depends on differences in the study populations (e.g., general population vs. athletes), type of exercise (aerobic vs. endurance) and methods used to measure exercise intensity (self-reported vs. objectively measured).

Intensive physical activity is generally contraindicated in overt hyper- and hypothyroidism, in order to prevent severe CV and muscular complications. Hyperthyroidism is associated with reduced exercise capacity and muscle weakness, which are reversible upon treatment [90]. Exertional dyspnea is common in hyperthyroid patients, primarily due to impaired oxygen diffusion, reduced ventilatory efficiency and respiratory muscle weakness [91]. Glycogen levels are low and deplete more rapidly during physical activity with increased lactate production [92]. These abnormalities normalize only after prolonged restoration of euthyroidism [93]. The effects of PA in patients with hypothyroidism have been explored in a limited number of studies, with inconsistent findings. While some evidence suggests that PA positively influences TH levels in untreated subclinical hypothyroidism, other studies have failed to demonstrate such an association [94]. In particular, it remains unclear whether changes in TH levels are induced by PA or are secondary to weight loss or changes in body composition.

Nevertheless, the benefits of regular PA in subclinical hypothyroidism extend beyond its potential impact on thyroid function. Indeed, exercise can help to manage several adverse outcomes of hypothyroidism, because it improves lipid profiles, alleviates mood disturbances, enhances sleep quality and improves perceived health [95]. Improved cardiorespiratory fitness is particularly valuable in elderly hypothyroid patients, for whom the long-term survival benefit of LT4 therapy remains uncertain [96]. Notably, adherence to PA programs can be challenging for hypothyroid individuals, as exercise intolerance has been reported in both treated and untreated patients [97]. This highlights the importance of supervised exercise programs to support patients’ engagement. Further research is essential to define the most effective and sustainable exercise strategies for this population. Although aerobic training is most frequently prescribed, endurance and postural exercises may be particularly beneficial for the elderly in counteracting age-associated sarcopenia.

## 10. Thyroid Dysfunction and Food Intake

Hyperthyroidism is associated with hyperphagic behavior, and LT4 replacement in hypothyroidism is linked to an increased hunger sensation [98]. These effects depend primarily on increased REE, but also on direct central actions of THs [99,100]. Indeed, T3 increases the expression of orexigenic peptides (NPY and AgRP) while decreasing the anorexigenic POMC in the arcuate nucleus. These effects are mediated by a complex mechanism likely involving both the TRα and TRβ isoforms. Indeed, the administration of the TRβ-specific agonist GC-1 to mice does not induce hyperphagia, whereas the administration of T3 does [101]. Furthermore, local knockdown of TRβ in the rat ventromedial paraventricular nucleus (VPN) induces hyperphagia and obesity [102], while TRβ knockout mice do not develop an obese phenotype [103].

Additional mechanisms influencing food intake involve the regulation of gastric emptying, gut motility and gallbladder function by THs. A largely unexplored area is whether THs influence food preferences. One study reported that patients with Graves’ disease exhibit hyperphagia, with a specific craving for carbohydrates, while protein and fat intake remained unchanged [99]. In a cohort of obese children and adolescents, higher FT4 levels were associated with a preference for protein- and fat-rich foods, although this association was not observed in controls [104]. Noteworthy, leptin, an adipokine involved in appetite suppression, stimulates TRH/TSH secretion [105] and upregulates D2 expression in skeletal muscle [106]. Taste and smell perception may also play a role, as both are reported to be impaired in hypothyroidism, which could explain the decreased appetite or even anorexia reported by some of these patients [107,108]. Interestingly, LT4 replacement therapy has been shown to improve taste perception [108]. Furthermore, a common polymorphism in type 2 bitter taste receptor (TAS2R42) has been linked to increased serum T4 levels in humans [109].

## 11. Resistance to Thyroid Hormone β (RTHβ)

RTHβ is a rare condition (estimated prevalence 1:20.000–40.000) caused by loss-of-function mutations in the THRB gene which encodes TRβ. These mutations impair binding with T3 or the recruitment/release of cofactors, while DNA binding and dimerization are preserved, resulting in altered transcriptional properties [1]. Most patients with this condition have heterozygous dominant-negative point mutations or small In/Dels located in the ligand-binding domain, whereas large deletions and mutations disrupting DNA binding cause the RTHβ phenotype only in the homozygous state, as reported in two families [110]. Therefore, the pituitary and the hypothalamus, which express the TRβ isoform, display partial insensitivity to THs, leading to TSH-dependent central hyperthyroidism with normal/high TSH and increased fT4/fT3 levels [111,112] (Table 1).

These increased levels of THs partially rescue hormone resistance in TRβ-expressing tissues (liver, kidney, retina and ear), while TRα-expressing tissues (heart, skeletal muscles, brain and guts), which retain normal sensitivity to THs, are thyrotoxic.

For this reason, RTHβ patients can exhibit both hypothyroid and hyperthyroid features [4]. The predominant myocardial expression of TRα explains the frequent cardiac involvement and the high prevalence of sinus tachycardia, AF and HF [113]. Other common clinical manifestations include goiter [114], attention-deficit/hyperactivity disorder (ADHD), anxiety [115,116], osteopenia, language or learning disabilities and failure to thrive in affected children [117]. Given that most affected patients are clinically euthyroid and often asymptomatic, RTHβ may act as a silent threat, potentially progressing toward more severe clinical manifestations if not properly identified and monitored. Indeed, a significantly increased risk of CVDs [118] and premature mortality [119] compared with the general population has been recently described in RTHβ patients [120]. Data from a UK cohort of adults with RTHβ have highlighted a modest but significant increase in cardiovascular risk, as measured by the QRISK3 algorithm, compared to matched healthy controls, and a prevalence of AF nearly double that of the general population [121]. Notably, 10% of these individuals had increased plasma NT-proBNP concentrations, supporting its routine measurement as a non-invasive marker of early cardiac involvement, with echocardiography recommended when levels are elevated. Echocardiographic alterations have been reported in RTHβ, with systolic and diastolic dysfunctions resembling those of untreated overt hyperthyroidism, while other parameters, such as left ventricular (LV) ejection fraction, systolic diameter and LV wall thickness, are comparable to that of the general population [90]. Moreover, RTHβ patients exhibit pro-atherogenic features resembling hypothyroidism, with a higher augmentation index (a marker of increased arterial stiffness) and elevated levels of LDL cholesterol [122] and triglycerides (mixed dyslipidemia) compared with euthyroid controls. Among the metabolic dysfunctions, we should also acknowledge that intramyocellular and intrahepatic lipid accumulation [123] cause MASLD [124], whereas data regarding insulin resistance in RTHβ are conflicting [123,125]; nevertheless, diabetes and high fasting glucose were highly prevalent (11%) in a large unselected Italian cohort of RTHβ patients [120]. REE is markedly elevated in both adults and children with RTHβ, accompanied by a 40% increase in energy intake and hyperphagia, especially in children [126], which may further contribute to dyslipidemia and insulin resistance.

According to the ETA guidelines, recommended treatments include beta-blockade alone or, rarely, in combination with triiodothyroacetic acid (TRIAC) therapy to control tachycardia and other thyrotoxic symptoms, such as anxiety, tremors and palpitations. However, the decision to treat patients with TRIAC should be made only after discussion with expert centers [1].

TRIAC is a natural acetic acid derivative of triiodothyronine that is produced in the liver and other peripheral tissues, through deamination and oxidative decarboxylation of the alanine side chain of THs [127,128]. The rationale for treatment is based on TRIAC’s preferential agonism for TR-β, leading to downregulation of TSH secretion in the pituitary, which, in turn, reduces TH levels and thyroid hyperplasia. This may alleviate thyrotoxic symptoms and goiter growth in RTHβ patients. Being selective for TR-β, TRIAC exerts minimal thyromimetic effects on tissues expressing TRα (e.g., heart, brain), while partially rescuing the function of TR-β mutants in the liver. The use of galenic TRIAC in RTHβ began around the 1980s [129], before the demonstration of a causal link between RTHβ with *THRB* mutation [130].

TRIAC effects on the prevention of adverse CV outcomes remain uncertain [1]. The same results are also lacking for lipid-lowering therapy and antihypertensives. An additional, unexplored aspect is whether standard treatment targets for LDL cholesterol, glycemia and blood pressure are appropriate for individuals with RTHβ, or whether more aggressive goals (e.g., LDL < 70 mg/dL = 1.8 mmol/L, blood pressure < 130/80 mmHg) should be considered, as in other high-risk populations (e.g., diabetes, hypertension, chronic kidney failure, etc.).

### Role of Lifestyle Modifications in RTHβ

Although there are currently no studies specifically addressing the influence of lifestyle changes in this population, the presence of dyslipidemia and insulin resistance suggests that lifestyle therapies warrant further investigation. Although individuals of all ages are likely to benefit from such interventions, younger patients and potentially children may represent the most promising target group, as early adoption of a healthy lifestyle habits may offer long-term protection of CV health, intervening before irreversible changes occur.

**Figure 2:**
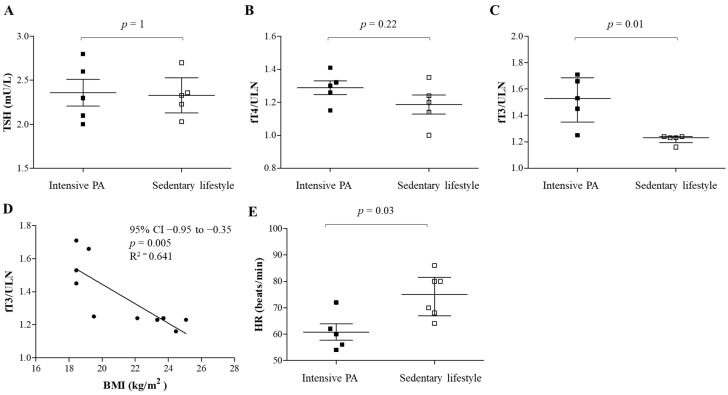
The influence of physical activity on a 35-year-old male affected by resistance to thyroid hormone β (RTHβ). (**A**–**C**) **Changes in TSH, fT4 and fT3** after discontinuing intensive physical activity and transitioning to a sedentary lifestyle. After discontinuation of physical activity, TSH and fT4 levels remained stable, while fT3 levels reduced and BMI increased. (**D**) **An inverse correlation between circulating fT3 and BMI**, demonstrating the relationship between thyroid function and body weight in RTHβ. (**E**) **Heart rate (HR) changes** following cessation of intensive physical activity, highlighting the impact of physical activity on cardiovascular and metabolic outcomes. fT4 is presented relative to the upper limit of the normal (ULN) to mitigate variations across different analytical methods. Legend: PA, physical activity; TSH, thyroid stimulating hormone; fT4, free thyroxine; fT3, free triiodothyronine; ULN upper limit of normal; HR, heart rate; BMI, body mass index.

An example of how lifestyle could influence the phenotypic expression of RTHβ is illustrated in Figure 2. This male patient harboring the p.V349L mutation in the THRB gene practiced high-level competitive cycling until the age of 35 years. After discontinuing agonistic PA, he gained 16 kg, with his body mass index (BMI) increasing from 18.4 to 24.4 kg/m^2^. His weight gain was associated with several metabolic consequences, including hepatic steatosis and dyslipidemia. His fasting hyperglycemia progressed to overt type 2 diabetes by age 43, with hypertension developing at 45. This clinical worsening was associated with a substantial decrease in circulating fT3 (Panel C), inversely correlating with BMI (Panel D). In contrast, TSH and fT4 levels remained stable (Panels A and B). This may reflect an altered T4-to-T3 conversion, possibly due to downregulation of D2 in the liver [131], or, alternatively, in skeletal muscles. Surprisingly, despite fT3 reduction, HR increased over time, suggesting that PA-induced modulation of adrenergic tone might play a more significant role in the cardiometabolic prevention of RTHβ, than the reduction in THs. This patient had two other affected relatives, one sedentary overweight sister and one brother who continued competitive cycling. Over time, his phenotype increasingly resembled that of his sister, who developed fasting hyperglycemia at the age of 36 and dyslipidemia and hypertension two years after menopause. In contrast, their brother remains metabolically healthy at 46 years of age, with optimal blood pressure, a normal resting HR, normal glycemia and favorable LDL levels (2.46 mmol/L). This case underscores that even though the weight gain did not result in a pathological BMI, the reduction in regular exercise triggered metabolic complications. This emphasizes that PA has the potential to modulate the phenotypic expression of RTHβ, even among genetically similar individuals.

Because patients with RTHβ have a higher risk of AF, we believe that the American Heart Association’s lifestyle recommendations for AF prevention [132] could also be safely extended to this population.

Moderate-intensity aerobic exercise (≥150 min per week), has been shown to improve cardiac function and quality of life without increasing arrhythmic risk. High-intensity interval training may also offer time-efficient benefits and improve cardiovascular fitness, although with uncertain effects on heart remodeling. Very intense or prolonged endurance activity (e.g., long-distance competitive sports) has been shown to increase the risk of AF. Therefore, individuals with RTHβ should be carefully evaluated and monitored in the case of high-intensity training to avoid potential adverse cardiac effects. Emotional factors, stress and anxiety are independent risk factors for arrhythmic events; thus, mind–body practices such as yoga or psychological support, if needed, could also be suggested to reduce symptomatic AF episodes [133].

In terms of diet, alcohol limitation should be prioritized, because ethanol consumption is a known risk factor for AF (and liver fat accumulation). Natural caffeine sources such as coffee, tea and chocolate are not contraindicated, while artificial stimulants (e.g., energy drinks) should be taken with caution, as they may trigger arrhythmic events even in normal individuals [134]. The Mediterranean diet, which is rich in antioxidants, unsaturated fats, Omega 3 and plant-based foods, might help in managing dyslipidemia, insulin resistance and hypertension in individuals with RTHβ.

Hepatoprotective diet supplements (silymarin, cynarine, curcumin) with antioxidant, anti-inflammatory and bile-stimulating properties may be useful adjunct therapies in the case of MASLD. Finally, smoking cessation remains an important component of cardiovascular risk reduction. Prospective studies are required to assess the long-term effectiveness of such interventions in this specific patient population. Attention should also be given to ensure optimal calcium intake through an appropriate diet or calcium/vitamin D supplements.

## 12. Resistance to Thyroid Hormone α (RTHα)

RTHα denotes the rare genetic condition resulting from a mutation in the *THRA* gene, which encodes TRα. Forty-one affected individuals have been reported so far [1]. The molecular mechanism underlying RTHα is similar to that described above for RTHβ, with loss-of-function mutations in the ligand-binding domain of *THRA* leading to impaired T3 binding or abnormal recruitment or release of cofactors (coactivators and corepressor), resulting in impaired transcriptional activity of TRs due to a dominant-negative action on the wild-type counterpart.

Clinical manifestations depend on the tissue distribution of TRα, which is primarily expressed in the brain, heart, skeletal muscles and gastrointestinal tract. These organs exhibit tissue-specific hypothyroidism [135] with variable severity, depending on the functional impairment of the TRα. Conversely, tissues expressing TRβ (pituitary, liver) remain normally sensitive to T3. Therefore, the pituitary–thyroid axis correctly functions and the biochemical features of RTHα are not easily recognizable. Indeed, thyroid function tests are characterized by normal TSH, low rT3, low–normal fT4 and normal or slightly elevated fT3, resulting in a high fT3/fT4 ratio [136] (Table 1). The increased fT3/fT4 ratio suggests altered TH metabolism, as also described in a TRαPV mice model which displayed markedly raised D1 expression in the liver. Alternatively, reduced D3 activity may also be a contributory factor, since its expression is regulated by TRα [135].

The clinical phenotype is variable, and ranges from clinically euthyroid patients to severe congenital hypothyroidism with delayed growth, macrocephaly, short disharmonic stature and neurodevelopmental and neuromotor problems [137,138]. Mild normocytic normochromic anemia is a common finding, with reduced red cell mass and hematocrit, but normal hematinic levels (iron, B12, folate, hemolytic indices and EPO concentrations) [139]. Only three cases with an elevated mean corpuscular volume (MCV) have been reported [140]. Other manifestations include muscle stiffness or hypotonia, associated with elevated creatine kinase, low REE and constipation due to slower gut motility and dry skin.

Although a specific CV risk has not been highlighted so far in patients with RTHα, it is worth noting that bradycardia and hypotension have been commonly described in this population. Moreover, abnormal heart structure, weakened contractility and disrupted sarcomere organization have been reported in zebrafish models of RTHα [141]. However, gross heart dysfunctions have never been reported in RTHα, except for in a single female patient carrying the C380fs mutation in the THRA gene, who developed hypertrophic obstructive cardiomyopathy and pericardial effusion [142]. Regarding CV risk factors, untreated adults may manifest major abdominal adiposity and a high LDL/HDL ratio [143].

According to guidelines, patients with RTHα should receive long-term levothyroxine therapy unless concerns about side effects or tolerability arise. Liothyronine has been administered only in one case [144].

Levothyroxine treatment for RTHα seems to be safe and beneficial for constipation, with linear growth in most patients, while the clinical responses regarding bradycardia, neurocognitive function, anemia, low IGF-1 and impaired GH response are variable among the reported cases.

Regarding cardiac outcomes, levothyroxine therapy has been shown to improve contractile function and HR without inducing secondary tachycardia [145,146,147]. The reason why the heart rate remains low is likely due to the reprogramming of the cardiac pacemaker function during embryonic development, with reduced expression of the Ryr2 calcium channel and Kcnh2 potassium channel [147]. This observation is reassuring regarding the safety of thyroxine therapy in RTHα, as doses sufficient to rescue hormone resistance can be administered without inducing tachycardia. Nevertheless, Kcnh2 downregulation has been associated with arrhythmia or sudden death; thus, cardiac monitoring of thyroxine-treated RTHα patients seems advisable [147].

### Role of Lifestyle Modifications in RTHα

There are currently no studies evaluating the impact of lifestyle interventions in patients with RTHα. A multidisciplinary strategy including neurology, neuropsychology, gastrointestinal intervention, hematology, dentistry, physical therapy, speech and language therapy and occupational therapy is recommended by guidelines [120].

Gastrointestinal and musculoskeletal issues are the main clinical manifestations that may benefit from non-pharmacological approaches. Constipation is a common feature of RTHα, usually caused by decreased peristaltic activity due to increased parasympathetic tone and reduced colonic motility [148]. Dietary recommendations include avoiding excessive fiber consumption and dehydration, which can exacerbate gastrointestinal dysmotility. A hypocaloric, fluid-rich diet, together with probiotic supplementation, may support gut function and help to prevent constipation or intestinal obstruction.

Skeletal muscle involvement is another hallmark of RTHα, with elevated CK levels, resembling hypothyroid myopathy. Regular physical activity is recommended to support neuromuscular development and functional autonomy and reduce fat accumulation. Aerobic exercise should be favored over anaerobic or resistance training to minimize the risk of muscle damage or even rhabdomyolysis. In children with persistent hypotonia and delayed motor milestones, treatment with levothyroxine (LT4) has shown greater benefits in improving muscle tone, motor development and linear growth, as compared with physical therapy alone [140]. Thus, the role of PA is mainly to reinforce the achievement of these milestones.

While LT4 improves musculoskeletal features and constipation, other features, including anemia, are not resolved [149]. Therefore, adequate dietary intake of folate, vitamin B12 and iron in RTHα patients may be even more important than in the general population. When this cannot be achieved with diet alone, supplementation should be considered. Finally, optimizing vitamin D and calcium intake is advisable to protect bone health.

## 13. Monocarboxylate Transporter 8 (MCT8) Defects

Mutations in the *SLC16A2* gene, which encodes MCT8, cause the rare X-linked genetic condition known as Allan–Herndon–Dudley Syndrome (AHDS) [1,6]. Pathogenic mutations include large deletions of one or more exons, smaller frameshift deletions, small In/dels, nonsense mutations causing a premature stop codon and missense mutations. Males are primarily affected, whereas female carriers may have negligible or no symptoms at all. MCT8 is expressed in many tissues, such as in those of the central nervous system, pituitary, liver, kidneys, skeletal muscles and thyroid gland. This transporter is involved in the influx of T3 into cells and the efflux of T4 in the thyroid gland and kidneys.

Being the only TH transporters expressed in the tanycytes and in the endothelial cells of the blood–brain barrier, MCT8 is crucial for transporting fT3 into the neurons [150]. Conversely, peripheral tissues are less dependent on MCT8, as several alternative thyroid hormone transporters are expressed (L-type amino acid transporters, OATP family, Na^+^-taurocholate cotransporting polypeptide and SLC17A4). This tissue distribution of THS transporters explains the biochemical profile of this syndrome, which is characterized by high serum fT3, low T4 and normal/slightly elevated TSH. Indeed, the impaired MCT8 function in the hypothalamus and pituitary may lead to TH insensitivity and unsuppressed TSH levels; conversely, T4 is trapped in the thyroid and in the kidney, resulting in upregulation of D1 and a further increase in T3 synthesis. Thus, patients with MCT8 deficiency are characterized by cerebral hypothyroidism, leading to severe neurodevelopmental impairment and intellectual disabilities. Paradoxically, fT3 accumulates in the blood, leading to different clinical sequelae secondary to chronic peripheral thyrotoxicosis. However, this condition usually remains misdiagnosed at birth because most children have good Apgar scores and a normal weight and head circumference. Moreover, these patients are false negatives in neonatal screening programs based on TSH assessment. The clinical phenotype appears later during infancy, with muscular hypotonia, poor head control and severely delayed cognitive and motor development. Most patients have spasticity or dystonia later in life, persistent primitive reflexes and seizures. They do not develop speech or the ability to sit or walk. Additional features are gastroesophageal reflux, feeding problems and constipation. Untreated patients have several thyrotoxic features, including tachycardia, systolic hypertension, raised REE, weight loss and osteoporosis. Life expectancy is severely impaired, with a median survival of 35 years, and approximately half of the most severely affected patients dying during childhood [6]. Sudden death, likely of cardiac origin, has been reported as a common cause of death. Cardiovascular function and nutritional status seem to play a role in mortality, since being underweight early in life is predictive of premature death [6].

There is no definitive cure, but several strategies have been investigated over time, including levothyroxine (alone or in combination with propylthiouracil) and the T3 analogues diiodothyropropionic acid and TRIAC, which can bypass MCT8 and enter cells through alternative transporters. In selected cases, in which the underlying molecular mechanism is the impairment of protein stability, with retained TH transport activity (e.g., MCT8delF501), 4-Phenylbutyrate has been shown to increase, in vitro, the cell surface expression of MCT8. The administration 4-Phenylbutyrate to MCT8 patients has been shown to improve thyroid function tests, but cause liver toxicity [151].

Among these compounds, only TRIAC has been studied in phase 2 clinical trials and a prospective cohort study. TRIAC therapy has proven to have beneficial CV and metabolic effects, in particular, improving tachycardia and systolic blood pressure, increasing body weight and decreasing markers of peripheral thyrotoxicosis (e.g., SHBG) [152] and fT3 [153].

While thyroid hormone analogues improve peripheral changes in MCT8 deficiency, unfortunately, no treatment for the associated neurological symptoms is available so far.

### Practical Considerations for Diet and Physical Exercise

Patients with MCT8 deficiency will benefit from supportive care such as physical therapy and nutritional support, as mortality is higher in underweight children [6]. In line with a patient-oriented approach, interdisciplinary care involving neuropediatrics, orthopedics, radiology, physiotherapy and speech and occupational therapy is essential. Indeed, parents and caregivers have identified neurodevelopmental improvement, along with outcomes such as body weight, motor dysfunction, dysphagia and gastroesophageal reflux, as key therapeutic priorities [154]. Nutrition is compromised due to impaired swallowing and the increased catabolic state caused by peripheral thyrotoxicosis. Supportive dietary care from professional dieticians is crucial to optimize nutritional status. Patients with major feeding problems can benefit from the placement of a feeding tube [155]. Moreover, flexible endoscopic evaluation of swallowing (FEES) could be considered to assess swallowing function and airway protection. Gastrostomy is another possible approach to support nutrition in patients presenting with underweight and recurrent pulmonary infections [156].

Levodopa/carbidopa and Botulinum toxin A have been proposed to treat spasticity, with uncertain results [157,158].

## 14. Selenoprotein Deficiency

Selenoprotein deficiency refers to reduced levels or function of one or more selenoproteins due to genetic mutations affecting the SECIS pathway [1,9,159]. Two main causes have been identified so far: biallelic pathogenic variants in *SECISBP2*, which encodes selenocysteine insertion sequence binding protein, or in the *TRU-TCA1-1* gene, which encodes selenocysteine transfer RNA [159].

Selenoproteins incorporate selenocysteine (Sec), which is essential for antioxidant defense and thyroid hormone metabolism. Key affected selenoproteins include deiodinases involved in thyroid hormone metabolism. Therefore, affected patients have a typical hormone profile characterized by slightly increased TSH, elevated fT4 and low T3 (Table 1).

Other important selenoproteins that are involved in this disorder include glutathione peroxidases (GPXs) and thioredoxin reductases (TXNRDs), which are crucial for detoxifying reactive oxygen species (ROS) and maintaining cellular redox balance, as well as selenoprotein N (SELENON), which is involved in muscle development and calcium homeostasis.

These abnormalities result in a multisystem phenotype, including immune dysfunction, growth retardation, male infertility, increased photosensitivity, sensorineural hearing loss and neurodevelopmental delays [160]. Patients affected by this condition are characterized by increased subcutaneous fat mass and reduced visceral fat associated with increased systemic insulin sensitivity. Moreover, SELENON deficiency causes axial and limb muscular dystrophy due to muscle weakness and hypotonia [8]. Increased prevalence of thoracic aortic aneurysms is an additional life-threatening feature of this syndrome [161].

Liothyronine replacement therapy corrects subnormal serum fT3 levels and improves linear growth [162]. Other possible treatments include oral selenium supplementation to restore plasma selenium concentrations [163], and antioxidants (e.g., alphatocopherol) to prevent oxidative damage [164]. Physical therapy can be applied as supportive care.

### Role of Lifestyle Modifications

In other muscle wasting disorders, such as muscular dystrophies and congenital myopathies, structured, personalized exercise regimens can help to preserve muscle function, minimize disuse atrophy and prevent muscle weakness from worsening [165]. Low-to-moderate-intensity aerobic activities like walking, cycling and swimming are not contraindicated, although they must be tailored to individual tolerance. Resistance exercises with light weights may strengthen core and proximal muscles, supporting posture and mobility. Stretching, high-resistance and repetitive movements (e.g., stair descents or deep squats) should be avoided to prevent muscle damage.

A well-balanced diet can play a supportive role in selenoprotein deficiency. A diet rich in fruits, vegetables, whole grains, legumes, nuts and olive oil will provide natural antioxidants, such as vitamins C and E, polyphenols and carotenoids, that help to counteract oxidative stress.

Additionally, to prevent arterial hypertension, which may contribute to vascular complications such as aortic dilatation, sodium restriction and sufficient intake of potassium and magnesium should also be prescribed. Beta-blockers, which are also used in collagenopathies like Marfan syndrome and Ehlers–Danlos syndromes, may be considered in order to maintain a resting heart rate of 60–70 bpm and keep the blood pressure below 120/80 mmHg. Adequate intake of vitamin D and calcium is crucial to support bone mineralization, which may be impaired by reduced mobility and muscle weakness, and specific supplements can be prescribed. Selenium supplementation, although not contraindicated in SECISBP2-related disorders, is usually ineffective [166]. Dietary supplements containing vitamins (A, C, E and carotenoids) which have antioxidant properties may also be beneficial for muscle health. Indeed, vitamin C is involved in collagen and carnitine synthesis, while retinol is involved in protein metabolism, collagen formation and lipid oxidation [167,168]. Another antioxidant, N-acetylcysteine (NAC, 70 mg/kg body mass), has positive effects on muscle force production during sustained fatiguing events. It is important to note that the appropriate dose to administer in patients with selenoprotein deficiency is unknown, but it is likely higher than that recommended for the general population. However, some evidence suggests that supraphysiological doses of vitamin C (1 g/day) and vitamin E (≥260 IU/day) may impair muscle adaptation to chronic exercise training in athletes [169]. Therefore, muscle performance and force should be monitored in these patients to determine the optimal dose for achieving antioxidant effects without compromising muscle function.

## 15. Limitations of the Study

Several limitations must be considered when interpreting the findings of this narrative review. Firstly, the conclusions are based primarily on observational studies and expert opinions, rather than randomized controlled trials. As a result, the strength of the evidence is limited, and definitive cause-and-effect relationships cannot be firmly established. Another key limitation is the lack of RCTs focused on lifestyle interventions, such as physical exercise and dietary modifications, in patients with rare thyroid disorders. While these interventions are known to influence cardiovascular and metabolic health, the absence of robust clinical trials in this population means that recommendations regarding their efficacy should be considered preliminary. Lastly, the genetic heterogeneity observed among patients with the same thyroid disorder further complicates generalization of the findings. While these patients share underlying genetic mutations, there is considerable variation in their clinical presentation, disease severity and response to treatment. This diversity highlights the need for personalized approaches to treatment that consider each patient’s characteristics. Future research should prioritize investigating these individual differences to create more tailored strategies for managing and preventing cardiometabolic risks in these populations.

## 16. Conclusions

Rare inherited disorders of thyroid hormone signaling are heterogeneous conditions, characterized by complex and multisystemic manifestations, including significant cardiometabolic implications. While advances in molecular diagnosis and clinical characterization have improved disease recognition, evidence-based strategies for long-term management remain limited, particularly in relation to lifestyle interventions.

Nutritional support and tailored physical activity are valuable tools in CV prevention and health maintenance in these patients.

While specific data are still lacking, especially for RTHα, MCT8 and SECISBP2 deficiencies, parallels drawn from similar neuromuscular and endocrine conditions suggest that structured exercise programs, antioxidant-rich diets, and early preventive strategies may be beneficial. Observational findings in RTHβ show that physical activity has significant effects on metabolic outcomes and thyroid hormone metabolism, implying that lifestyle can considerably influence the phenotypic expression of this syndrome. Prospective research and randomized, multicenter controlled trials are critical for confirming these assumptions and guiding future therapeutic practice. Physicians should consider including tailored exercise regimens, nutritional counseling and cardiovascular surveillance in routine treatment, especially in younger people, where early intervention may provide long-term protection.

## Figures and Tables

**Table 1:** Clinical, biochemical and targetable manifestations of genetic disorders of thyroid hormone signaling.

Disorder	Resistance to Thyroid Hormone Beta (RTHβ)	Resistance to Thyroid Hormone Alpha (RTHα)	Monocarboxylate Transporter 8 (MCT8) Deficiency	Selenoprotein Deficiency
**Gene**	*THRB*	*THRA*	*SLC16A2*	*SECISBP2* or *TRU-TCA 1-1*
**Free T4**	High	Low–normal or low	Low–normal or low	High
**Free T3**	High	High–normal or high	Usually high or high–normal	Low or normal
**Reverse T3**	High	Normal or low	Low	High
**TSH**	Normal or high	Normal (or mildly raised)	Normal (or mildly raised)	Normal
**Main cardiovascular** **manifestations**	Tachycardia Atrial fibrillation Cardiac insufficiency	Bradycardia Low BP	Tachycardia Systolic hypertension	Thoracic aortic aneurysm
**Main metabolic** **manifestations**	Failure to thrive High REE Dyslipidemia MASLD Insulin resistance Osteopenia	Low REE	High REE Osteopenia/osteoporosis	Increased fat mass Increased systemic insulin sensitivity
**Other relevant or targetable manifestations**	Anxiety, behavioral disorders, neurocognitive impairment	Anemia, neurocognitive impairment, constipation	Neurocognitive impairment, hypotonia/dystonia, gastroesophageal reflux, feeding problems, constipation	Photosensitivity Axial and limb muscular dystrophy Male infertility
**Useful lifestyle/dietary** **interventions**	Mediterranean diet, optimize vitamin D and calcium intake Mind–body practices and psychological support Antioxidants Silymarin, cynarine, curcumin for MASLD	Regular aerobic exercise Hypocaloric diet Optimize vitamin D and calcium intake Optimize iron, vitamin B12 and folate intake Probiotics and liquid-rich diet	Optimize vitamin D and calcium intake Psychological and physiotherapeutic support Dietologist/dietician support to avoid malnutrition, feeding tubes, gastrostomy Probiotics	Optimize vitamin D and calcium intake Antioxidants Physiotherapeutic support/regular exercise Hypo-sodium diet
**Useful treatments** [1]	TRIAC, cardioselective beta-blockers, anti-arrhythmic drugs	L-T4	TRIAC, cardioselective beta-blockers, anti-emesis drugs, spasmolytic and/or anti-cholinergic drugs, anti-constipation remedies	Antioxidants UV skin protection

Legend: L-T4, levothyroxine; UV, ultraviolet; MASLD, Metabolic Dysfunction-Associated Steatotic Liver Disease; REE, resting energy expenditure; TRIAC, Triiodothyroacetic acid; BP, blood pressure.

## Data Availability

Data sharing is not applicable to this article. The original contributions presented in Figure 2 are included in the article.

## Abbreviations

The following abbreviations are used in this manuscript:

AF	Atrial fibrillation
AgRP	Agouti-related protein
BAT	Brown adipose tissue
BP	Blood pressure
CV	Cardiovascular
D1	Type 1 deiodinase
D2	Type 2 deiodinase
D3	Type 3 deiodinase
DIOs	Iodothyronine deiodinases
3, 5-DIT	Diiodotyrosine
HF	Heart failure
HPTA	Hypothalamus–pituitary–thyroid axis
HR	Heart rate
IRD	Inner-ring deiodination
LT-4	Levothyroxine
MACEs	Major CV events
MASLD	Metabolic Dysfunction-Associated Steatotic Liver Disease
MCT8	Monocarboxylate Transporter 8
3′-MIT	Monoiodotyrosine
NIS	Sodium/iodide symporter
NPY	Neuropeptide Y
ORD	Outer-ring deiodination
PA	Physical activity
POMC	Proopiomelanocortin
PVN	paraventricular nucleus
RAAS	Renin–angiotensin–aldosterone system
REE	Resting energy expenditure
RTHα	Resistance to thyroid hormone α
RTHβ	Resistance to thyroid hormone β
SECISBP2	Selenocysteine Insertion Sequence-Binding Protein 2
SELENON	Seloprotein N
SLC16A2	Solute Carrier Family 16 Member 2
T3	Triiodothyronine
T4	Thyroxine
THs	Thyroid hormones
TPO	Thyroid peroxidase
TRH	Thyrotropin-releasing hormone
TRHR	Thyrotropin-releasing hormone receptor
TRIAC	3,3′,5-Triiodothyroacetic Acid
TRs	Thyroid hormone receptors
TRα	Thyroid hormone receptor α
TRβ	Thyroid hormone receptor β
TSH	Thyroid stimulating hormone
UV	Ultraviolet
VMH	Ventromedial nucleus of the hypothalamus
WAT	White adipose tissue
